# Experimental and mathematical analysis of cAMP nanodomains

**DOI:** 10.1371/journal.pone.0174856

**Published:** 2017-04-13

**Authors:** Christian Lohse, Andreas Bock, Isabella Maiellaro, Annette Hannawacker, Lothar R. Schad, Martin J. Lohse, Wolfgang R. Bauer

**Affiliations:** 1 Institute of Pharmacology and Toxicology, University of Würzburg, Würzburg, Germany; 2 Computer Assisted Clinical Medicine, University of Heidelberg, Heidelberg, Germany; 3 Comprehensive Heart Failure Center, University of Würzburg, Würzburg, Germany; 4 Department of Medicine I, University Hospital Würzburg, Würzburg, Germany; University of Oslo, NORWAY

## Abstract

In their role as second messengers, cyclic nucleotides such as cAMP have a variety of intracellular effects. These complex tasks demand a highly organized orchestration of spatially and temporally confined cAMP action which should be best achieved by compartmentalization of the latter. A great body of evidence suggests that cAMP compartments may be established and maintained by cAMP degrading enzymes, e.g. phosphodiesterases (PDEs). However, the molecular and biophysical details of how PDEs can orchestrate cAMP gradients are entirely unclear. In this paper, using fusion proteins of cAMP FRET-sensors and PDEs in living cells, we provide direct experimental evidence that the cAMP concentration in the vicinity of an individual PDE molecule is below the detection limit of our FRET sensors (<100nM). This cAMP gradient persists in crude cytosol preparations. We developed mathematical models based on diffusion-reaction equations which describe the creation of nanocompartments around a single PDE molecule and more complex spatial PDE arrangements. The analytically solvable equations derived here explicitly determine how the capability of a single PDE, or PDE complexes, to create a nanocompartment depend on the cAMP degradation rate, the diffusive mobility of cAMP, and geometrical and topological parameters. We apply these generic models to our experimental data and determine the diffusive mobility and degradation rate of cAMP. The results obtained for these parameters differ by far from data in literature for free soluble cAMP interacting with PDE. Hence, restricted cAMP diffusion in the vincinity of PDE is necessary to create cAMP nanocompartments in cells.

## Introduction

Cyclic nucleotides such as cyclic adenosine monophosphate (cAMP) act as second messengers, transducing extracellular stimuli into intracellular signals and leading to various effects inside the cell. In general, the cAMP concentration is assumed to be homogeneous throughout the entire cell body. However, more recent findings suggest that second messenger signaling displays an intriguing complexity requiring a more complex intracellular distribution to achieve signaling specificity. These observations gave rise to the concept of cAMP compartmentation—intracellular compartments with different concentrations of cAMP [1]. Key roles in the formation of cAMP compartments are attributed to the cAMP production by adenylyl cyclases (AC) and the degradation by phosphodiesterases (PDE) [2–16].

Although first reports on cAMP compartments in single living cells have been published several decades ago [17–19], the molecular details of cAMP compartmentation are still unclear. In principle, several mechanisms might contribute to local cAMP gradients. These are: local production of cAMP by membrane-bound ACs, cAMP buffering by regulatory PKA subunits, cAMP export by multidrug resistance proteins, restricted cAMP diffusion by yet undefined physical barriers, cell shape, and local cAMP degradation by PDEs (reviewed in [20]). Most prominently, local degradation of cAMP by PDEs has been shown to be responsible for experimentally observed cAMP microdomains, as inhibition of PDEs eliminated cAMP gradients within a cell [17, 21–24]. In addition, many computational studies have suggested that PDEs play an essential role in shaping cAMP gradients within a cell (reviewed in [20]). However, two important points should be considered. First, all reported studies to date focus on the description of rather large, so-called microdomains of cAMP, e.g. cAMP gradients between membrane compartments and cytosolic compartments. Second, all computational studies indicating a role of PDEs in shaping cAMP gradients have used either artificially high turnover rates, slow cAMP diffusion or unphysiologically high enzyme concentrations (e.g. [2, 25, 26]). Hence, although experimental data supporting the existence of cAMP microdomains have been obtained by many groups and the involvement of PDEs is well documented, there are conflicting data as far as the molecular mechanisms of cAMP compartmentation are concerned. In this study we apply an interdisciplinary biophysical approach to study cAMP compartments surrounding individual PDE molecules in intact cells. By using fusion proteins of cAMP FRET-sensors and PDEs we measure cAMP concentrations in the direct vicinity of a single PDE molecule in living cells. We find that the cAMP concentration next to a single PDE molecule is undetectable by our FRET-sensor (<100nM) even when the cells are stimulated with a maximal concentration of the *β*-adrenergic agonist isoproterenol. Interestingly, this “shielding” of the cAMP sensor from cAMP by the adjacent PDE molecule is partially persistent in diluted, crude cytosolic preparations. This renders cAMP diffusion, PDE activity, and PDE clustering as the most prominent and sufficient mechanisms to account for cAMP compartmentation by PDEs.

To establish a theoretical framework based on these experimental data, we develop analytically-solvable, diffusion-reaction equations to describe cAMP nanocompartments biophysically. By deriving estimates for the interrelation of diffusive mobility of cAMP and cAMP degradation rates, the interdisciplinary experimental and modeling approach applied here narrows down the possible mechanisms for cAMP compartmentation to three most important factors, i.e. restricted cAMP diffusion, PDE catalytic activity, and, to some extent, PDE clustering. Taken together, our data indicate that cAMP diffusion must be significantly slower or more heterogeneous than previously reported to allow for the observation of cAMP nanocompartments around a single PDE molecule in intact cells.

## Methods

### FRET measurements in living cells

Live single-cell FRET imaging was carried out in HEK-TsA cells as described previously [27]. In brief, HEK-TsA cells were plated on glass coverslips in 6 well-plates and transiently transfected either with the fluorescent cAMP sensor Epac1-camps, a direct fusion between the sensor and PDE4A1, termed Epac1-camps-PDE4A1 or its catalytically-impaired derivative Epac1-camps-PDE4A1(D352A).

48 h after transfection, cells were mounted in an imaging chamber and FRET ratios were measured in single cells in real time before and after the addition of the *β*-adrenergic agonist isoproterenol (1*μ*M) and the PDE4-specific inhibitor rolipram (1*μ*M). Ratiometric FRET imaging was performed using an upright epifluorescence microscope (Axio Observer, Zeiss, Germany) equipped with a water-immersion objective (63X/1.1 numerical aperture), a xenon lamp coupled to a monochromator (VisiView, VisiChrome, Germany), filters for CFP (436/20, 455LP dichroic) and YFP (500/20, 515LP dichroic) excitation, a beam splitter (DualView, Photometrics, Germany) with a 505LP dichroic mirror and emission filters for CFP (480/30) and YFP (535/40), and an electron-multiplied charge coupled device (EMCCD) camera (Evolve 512, Photometrics, Germany). CFP and YFP images upon CFP excitation were captured every 5s with 50ms illumination time. FRET was monitored in real-time with the MetaFluor 5.0 software (Molecular Devices) as the ratio between YFP and CFP emission. The YFP emission was corrected for direct excitation of YFP at 436nm and the bleedthrough of CFP emission into the YFP channel as previously described [28]. Images were analyzed utilizing the Graph Pad Prism 6.0 software (GraphPad Software, La Jolla, California, USA).

### In vitro measurements of cAMP-induced changes of FRET ratios

HEK-TsA cells were transfected with the cDNAs encoding either Epac1-camps, or the fusion proteins Epac1-camps-PDE4A1, or Epac1-camps-PDE4A1 (D352A), or PDE4A1 using calcium phosphate precipitation. 48h after transfection cells were harvested and ca. 1 × 10^7^ cells were resuspended in 300*μ*l of 10mM TRIS-HCl, 10mM MgCl_2_ buffer (pH7.4) containing 1mM PMSF and protease inhibitors (20*μ*g/mL soybean trypsin and 60*μ*g/mL benzamidin). Cells were broken by two 10s bursts of an Ultraturrax device. Cell debris and nuclei were removed by centrifugation (1,000xg, 5min, 4°C) and the supernatant was centrifuged again (100,000xg, 30min, 4°C) to yield the cytosolic fraction. For FRET experiments, 80 − 120*μ*l of the cytosol were diluted with buffer ad 600*μ*l. Fluorescent spectra of the cytosolic fractions between 460nm and 550nm were recorded upon illumination with 436nm before and after addition of increasing concentrations of cAMP using the LS50B spectrometer (PerkinElmer Life Sciences, Waltham, Massachusetts, USA). To ensure equal sensor concentration during measurements, all cytosol preparations were adjusted to the same YFP emission intensity (535nm upon direct illumination at 500nm). To quantify the effects of global PDE activity, two cytosolic fractions expressing either Epac1-camps or PDE4A1 were mixed in a manner that recapitulates the expression of Epac1-camps-PDE4A1. The amount of the Epac1-camps cytosol was adjusted to the same YFP emission intensity measured in cytosolic fractions of Epac1-camps-PDE4A1. The amount of the PDE4A1 cytosol was then adjusted to the same PDE activity as measured with Epac1-camps-PDE4A1. Concentration-effect curves were generated by calculating the 535nm/480nm FRET emission ratios at different cAMP concentrations. Data points were fitted with a three-parameter logistic equation using Graph Pad Prism 6.0 (GraphPad Software, La Jolla, California, USA). Data were then normalized to the lower (absence of cAMP; set to 0%) and upper plateau (saturating concentrations of cAMP, set to 100%) of the concentration-effect curve.

## Results

### Experimental determination of cAMP-protected domains

**Fig 1:**
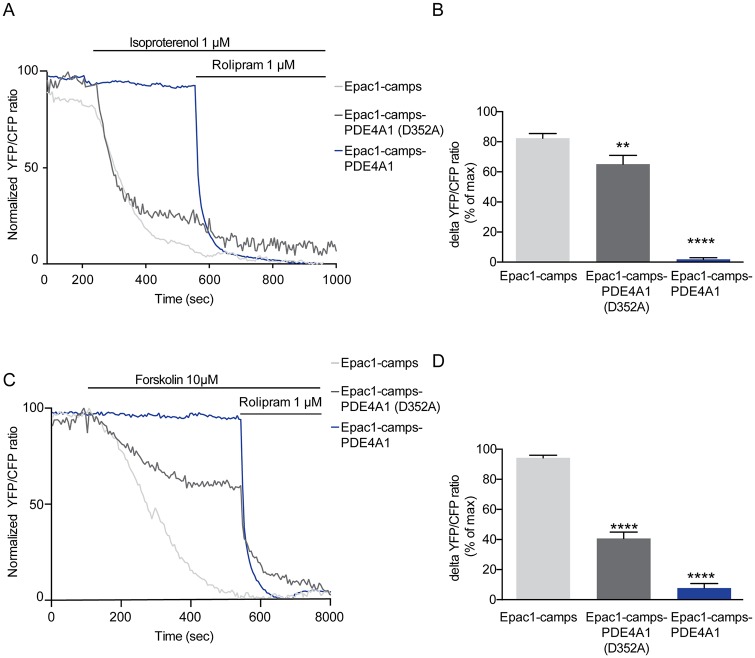
Fusion of PDE4A1 to Epac1-camps generates cAMP nanodomains in living cells. (a) and (c) Representative traces of the normalized FRET (YFP/CFP) ratio of the indicated constructs. Increases of cAMP were obtained by activation of endogenous *β*-receptors by isoproterenol (a) or upon stimulation with the direct activators of adenylyl cyclase forskolin (c) and (d) Amplitude of the cAMP response elicited by isoproterenol (b) or forskolin (d) expressed as a percentage of maximal stimulation induced by rolipram. Data are shown as means ± s.e.m. of at least 6 independent experiments. Differences vs. Epac1-camps were statistically significant by one-way ANOVA followed by Bonferroni post hoc-test **, P < 0, 001 ****, P < 0,00001.

To measure the cAMP concentration in direct vicinity of a single PDE molecule, we expressed fusion proteins of the cAMP FRET-sensor Epac-1-camps [29] and PDE4A1 [27] in HEK-TsA cells. The live cell FRET experiments carried out here (Fig 1) show that no change in the FRET ratio was recorded with isoproterenol when the sensor was fused to an active PDE (Epac1-camps-PDE4A1). In contrast, activation of endogenous *β*-receptors upon isoproterenol stimulation induced a nearly maximal decrease in the FRET ratio in cells expressing only the cAMP FRET-sensor Epac1-camps. No further decrease in the FRET ratio was recorded upon inhibition of endogenous PDE4s with rolipram. The protection of Epac1-camps seen in the presence of an active PDE is specific to the local PDE activity because first, it was partially lost upon expression of a fusion protein containing a catalytically-impaired PDE mutant (Epac1-camps-PDE4A1(D352A)), and second, the protection was completely lost, if the PDE was blocked by the PDE4 specific inhibitor rolipram. These experiments indicate that the cAMP concentration in direct vicinity of an active PDE is below the detection limit of the sensor [29]. The effect of isoproterenol on the different sensor proteins was mimicked also if more persistent cAMP signals were elicited upon direct activation of adenylyl cyclases by forskolin (Fig 1c and 1d). Overall these data suggest that PDE may act as a “sink” and thereby creates a local cAMP minimum, regardless of the stimulus used to elicit a cAMP signal.

**Fig 2:**
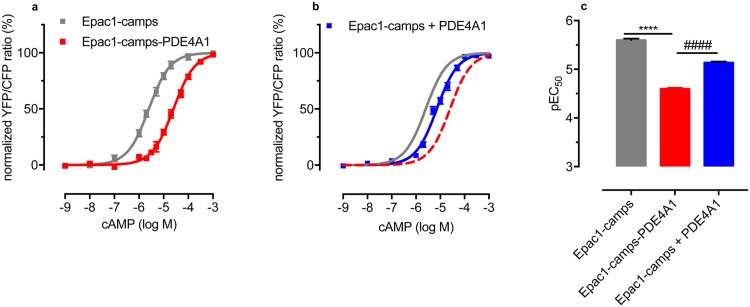
Local PDE activity creates a cAMP gradient in cytosolic fractions. Concentration-effect curves of cAMP-induced changes of the FRET ratios of the cAMP sensors Epac1-camps (grey curve) and Epac1-camps-PDE4A1 (red curve) in soluble cytosolic preparations of transiently transfected HEK-TsA cells. The presence of PDE activity in the fusion protein leads to a loss of apparent affinity of the FRET-sensor for cAMP (rightward shift of the concentration-effect curve). (b)Separate expression of equal amounts of Epac1-camps and PDE4A1 (blue curve) leads to a right-shift of the concentration-effect-curve, albeit to a lesser extent than the fusion protein. The curves for Epac1-camps and Epac1-camps-PDE4A1 are shown for comparison as grey and red dashed lines, respectively. (c) Apparent affinities (pEC_50_) of Epac1-camps, Epac1-camps-PDE4A1, and Epac1-camps + PDE4A1 for cAMP are 5.60 ± 0.03(= 2.5*μ*M), 4.60 ± 0.02(= 25*μ*M), and 5.14 ± 0.02(= 7.2*μ*M), respectively. This indicates that the cAMP concentration in close proximity to the PDE is less than the concentration of the surrounding solution. Experiments were carried out in 10mM TRIS, 10mM MgCl_2_, pH7.4 and FRET changes were recorded upon addition of increasing concentrations of cAMP. Data are normalized to the maximum change of the FRET ratio at saturating concentrations of cAMP (= 100%) and the basal FRET ratio in the absence of cAMP (= 0%), respectively. The slope of all curves is not significantly different from n = 1 (P = 0.53, P = 0.37, P = 0.80 for Epac1-camps, Epac1-camps-PDE4A1, and Epac1-camps + PDE4A1, respectively). Data are means ± s.e.m. of at least three independent experiments carried out with 3-5 repetitions. ****, ####, (P < 0.0001) according to one-way ANOVA with Tukey’s multiple comparison test.

To check if a local cAMP minimum around a PDE persists even upon destruction of the cellular architecture, we conducted biochemical in vitro FRET experiments in cytosolic fractions of transfected HEK-TsA cells (S1 Fig). In contrast to the experiments in intact cells (c.f. Fig 1) the fusion protein Epac1-camps-PDE4A1 did respond to cAMP. However, the sensitivity was significantly lower than that of Epac1-camps (note the rightward shift of the concentration-effect curve in Fig 2a). The observed 10-fold decrease in apparent cAMP affinity is due to PDE activity as the catalytically-impaired construct Epac1-camps-PDE4A1 (D352A) had the same apparent affinity for cAMP as Epac1-camps (S2 Fig). To exclude that the rightward-shift of the cAMP concentration-effect curve is due to a global elevation of PDE activity, we expressed Epac1-camps and PDE4A1 as separate proteins. To achieve a 1:1 stoichiometry of the two proteins, we mixed the respective cytosols in amounts which matched (1) the concentration of FRET-sensor as determined by YFP emission intensity and (2) the degree of PDE activity as determined by real-time FRET measurements (S3 Fig). Usually, the ratio between the cytosolic fractions expressing Epac1-camps and PDE4A1, respectively, was 1:2 to achieve 1:1 stoichiometry. Under these conditions, the cAMP concentration-effect curve was significantly shifted to the right (Fig 2b), however, the rightward-shift was clearly not as pronounced as with the fusion protein Epac1-camps-PDE4A1 (Fig 2c). These data demonstrate that PDE activity can create a local cAMP minimum.

### Mathematical analysis of cAMP degradation on a single molecule level

**Fig 3:**
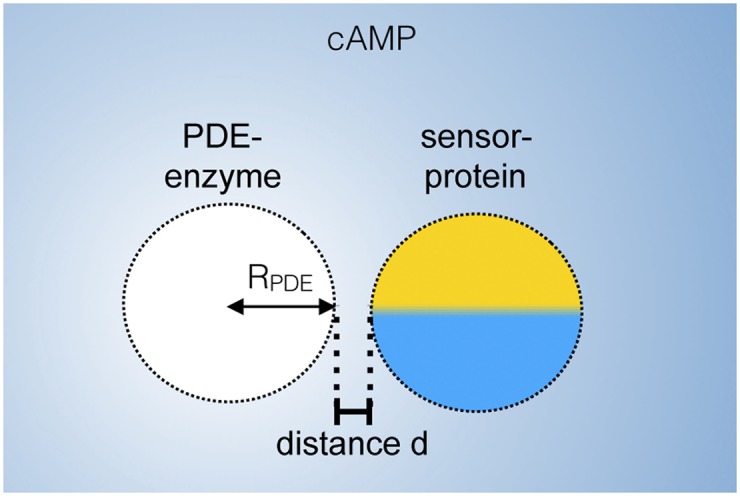
Schematic description of the experimental design used to measure cAMP nanocompartments on a single molecule resolution. The PDE molecule is modeled as an absorbing sphere of radius *R*_PDE_ and the sensor protein attached adjacent (distance *d*) is used to measure the cAMP concentration.

Based upon the experimental data presented here, we develop a biophysical model for cAMP degradation on a single molecule level (Fig 3). The sensor mechanism itself depends on the Förster / fluorescence resonance energy transfer (FRET), yielding the cAMP binding ratio *S* of the sensor molecule. This ratio depends on the cAMP concentration at the sensor molecule,

$$\begin{array}{c} S=\frac{\rho_{Sensor-cAMP}}{\rho_{Sensor}+\rho_{Sensor-cAMP}}=\frac{\rho_{cAMP}}{K_{D}+\rho_{cAMP}}{|}_{r=R_{\text{PDE}}+d}\;, \end{array}$$ (1)

where *K*_*D*_ is the dissociation constant of the sensor protein, *R*_PDE_ the radius of the spherical assumed PDE, and, hence, *R*_PDE_ + *d* is the position of the sensor molecule, when the center of the PDE is considered as reference.

To obtain the cAMP concentration $\rho (\vec{r},t)$ around the PDE we assume free and homogeneous diffusion. We further assume isotropic diffusion around the PDE, and isotropic reaction conditions on the surface of the PDE, i.e. only a radial dependence of *ρ* remains when expressed in spherical coordinates $\vec{r}=\left(r,\theta ,\phi \right)$, i.e. $\rho \left(\vec{r},t\right)=\rho \left(r,t\right)$. This symmetry allows to write Fick’s diffusion laws as

$$\begin{array}{ccc} \partial_{t}\rho \left(r,t\right) & = & \nabla \vec{j}\left(r,t\right)\\ & = & Dr^{-2}\partial_{r}\left(r^{2}j_{r}\left(r,t\right)\right)\\ j_{r}\left(r,t\right) & = & -D\partial_{r}\rho \left(r,t\right) \end{array}$$ (2)

with diffusion coefficient *D* and diffusive flow density $\vec{j}$. As only the radial component of this flow is of relevance, we omit the subscript *r*, i.e. *j*_*r*_ = *j*. We also consider solely steady state conditions, i.e. the cAMP concentration is stationary ∂_*t*_*ρ* = 0, and, hence,

$$\begin{array}{c} r^{2}j\left(r\right)\equiv \;\text{const.}\;. \end{array}$$ (3)

This is justified as variations from the steady state are equilibrated within nanoseconds. The absorption rate, quantified by the flux $j\left(R_{\text{PDE}}\right)4\pi R_{\text{PDE}}^{2}$ through the reactive protein surface, is assumed to follow a Michaelis-Menten kinetic [30],

$$\begin{array}{c} j\left(R_{\text{PDE}}\right)4\pi R_{\text{PDE}}^{2}=-\frac{k_{cat}}{\left(K_{m}+\rho \right)}\rho {|}_{r=R_{\text{PDE}}}\;, \end{array}$$ (4)

where *k*_*cat*_ is the maximum degradation rate of a single PDE molecule and *K*_*m*_ the Michaelis-Menten constant which is defined as the substrate concentration at which the degradation rate is half of *k*_*cat*_. As we consider only one reaction center, the cAMP concentration reaches a constant asymptotic value far away from the center

$$\begin{array}{c} \lim_{r\to \infty }\rho \left(r\right)=\rho_{0} \end{array}$$ (5)

From Eqs (3) and (5) directly follows

$$\begin{array}{c} \rho =\rho_{0}(1-A\frac{R_{\text{PDE}}}{r})\;, \end{array}$$ (6)

where the constant *A* is determined from the boundary condition on the protein surface—describing the enzyme kinetics of the PDE (Eqs (2) and (4)),

$$\begin{array}{ccc} D\partial_{r}\rho \left(r\right){|}_{r=R_{\text{PDE}}} & = & \frac{1}{4\pi R_{\text{PDE}}^{2}}\frac{k_{cat}\rho }{K_{m}+\rho }{|}_{r=R_{\text{PDE}}}\\ DA\rho_{0}\frac{1}{R_{\text{PDE}}} & = & \frac{1}{4\pi R_{\text{PDE}}^{2}}\frac{k_{cat}\rho_{0}\left(1-A\right)}{K_{m}+\rho_{0}\left(1-A\right)} \end{array}$$ (7)

Re-scaling of the cAMP concentration by the Michaelis-Menten constant *ρ* → *c* = *ρ*/*K*_*m*_ and introducing the dimensionless absorptive action

$$\begin{array}{c} \eta =\frac{k_{cat}}{4\pi R_{\text{PDE}}DK_{m}} \end{array}$$ (8)

allows to rewrite Eq (7)

$$\begin{array}{c} Ac_{0}=\eta \frac{c_{0}\left(1-A\right)}{1+c_{0}\left(1-A\right)} \end{array}$$ (9)

This equation has two solutions for A,

$$\begin{array}{c} A_{1,2}=\frac{\left(1+\eta +c_{0}\right)\pm \sqrt{{\left(1+\eta +c_{0}\right)}^{2}-4c_{0}\eta }}{2c_{0}}\;, \end{array}$$ (10)

but only

$$\begin{array}{c} A=\frac{\left(1+\eta +c_{0}\right)-\sqrt{{\left(1+\eta +c_{0}\right)}^{2}-4c_{0}\eta }}{2c_{0}} \end{array}$$ (11)

is of physical relevance, since only this solution satisfies the limiting constraint in the absence of absorption lim_*η* → 0_, where concentrations approach their asymptotic values far away from the reaction center *ρ* → *ρ*_0_, or *c* → *c*_0_ (see Eq (6)). From this we finally obtain the solution for the cAMP concentration around the PDE molecule

$$\begin{array}{c} c=c_{0}(1-\frac{\left(1+\eta +c_{0}\right)-\sqrt{{\left(1+\eta +c_{0}\right)}^{2}-4c_{0}\eta }}{2c_{0}}\frac{R_{\text{PDE}}}{r})\;. \end{array}$$ (12)

Note that the dimensionless absorptive action *η* in Eq (8) is an important parameter which describes the capability of a reactive center to create a concentration sink of cAMP. It relates the time scale of cAMP degradation *k*_*cat*_ to that of its diffusive mobility *D*. In addition it also scales spatial dimensions to the extension of the reactive center *R*_PDE_.

### Requirements for compartmentalization

**Fig 4:**
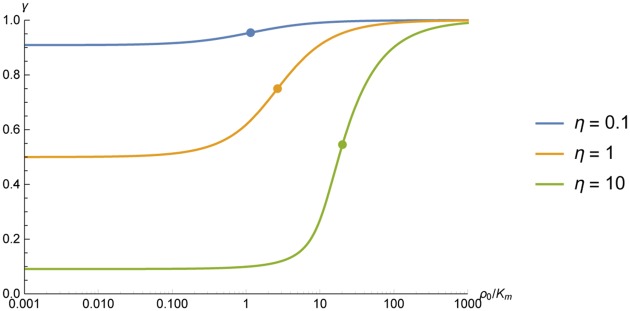
Depth *γ* of the nanocompartments formed by a single PDE molecule as a function of the cAMP concentration *c* for different values of absorptive action *η*. The concentration *c* is given in multiples of the Michaelis-Menten constant of the PDE $c=\frac{\rho }{K_{m}}$, the absorptive action *η* is defined as $\eta =\frac{k_{cat}}{4\pi R_{\text{PDE}}DK_{m}}$. The deepest compartments are found in the limit *c* → 0, where $\gamma =\frac{1}{1+\eta }$. When the cAMP concentration is increased (*c* → ∞), the compartments get flooded and disappear due to saturation of the PDE. Dots indicate the concentration at which the compartment is half way flooded and are found at $c_{1/2}=\frac{{\left(1+\eta \right)}^{2}}{1+\eta /2}$.

To quantify the capability of a PDE molecule to generate a nanocompartment we define its depth and width as follows. The width *δ* is the distance from the center of absorption, at which cAMP concentration reaches the average of its maximum *c*_0_ = *c*(∞) and minimum *c*(*R*_PDE_) value,

$$\begin{array}{c} c\left(\delta \right)=\frac{c_{0}+c\left(R_{\text{PDE}}\right)}{2}\Rightarrow \delta =2R_{\text{PDE}}\;, \end{array}$$ (13)

and correspondingly the depth *γ* as the ratio between the minimum and maximum concentration of cAMP,

$$\begin{array}{c} \gamma =\frac{c(R_{\text{PDE}})}{c_{0}}=\frac{c_{0}-1-\eta +\sqrt{{\left(1+\eta +c_{0}\right)}^{2}-4c_{0}\eta }}{2c_{0}}\;, \end{array}$$ (14)

i.e. *γ* = 1 when there is no concentration gradient, and *γ* = 0 when there is no cAMP near the PDE molecule. The width of the nanocompartment depends on the spatial extension of the absorbing enzyme (*δ* = 2*R*_PDE_), which implies that a single molecule can only generate a nanocompartment of roughly twice the size of the absorbing enzyme. The depth *γ* depends on the absorptive action *η*, and, which is important, on the concentration *c*_0_ of the surrounding cAMP (see Fig 4). The deepest compartments are found at low concentrations *c*_0_ → 0 and vice versa the compartments disappear (*γ* → 1) for high values of *c*_0_ → ∞. This is due to the saturation of PDE activity that starts at concentrations higher than the Michaelis-Menten constant *ρ*_*cAMP*_ = *K*_*m*_, i.e. *c*_0_ = 1. Once the PDE approaches its highest performance, the compartment is filled and the depth decreases when further increasing the cAMP concentration.

The above mentioned relationships may be quantified (see Fig 4). In the limit for vanishing external cAMP concentration *c*_0_ → 0, one gets

$$\begin{array}{c} \lim_{c_{0}\to 0}\gamma =\lim_{c_{0}\to 0}(\frac{c(r=R_{\text{PDE}})}{c_{0}})=\frac{1}{1+\eta } \end{array}$$ (15)

and in the other limit *c* → ∞

$$\begin{array}{c} \lim_{c_{0}\to \infty }\gamma =\lim_{c_{0}\to \infty }(\frac{c(r=R_{\text{PDE}})}{c_{0}})=1 \end{array}$$ (16)

This implies that the concentration, *c*_1/2_, at which the compartment is half way flooded, i.e. when $\gamma \left(c_{1/2}\right)=\frac{1}{2}\left(\gamma \left(c\to 0\right)+\gamma \left(c\to \infty \right)\right)$, is

$$\begin{array}{c} c_{1/2}=\frac{{\left(1+\eta \right)}^{2}}{1+\eta /2} \end{array}$$ (17)

These points are marked on the curves in Fig 4. Note that for vanishing enyme activity (*η* → 0) this concentrations approaches the Michaelis-Menten constant as *c*_0_ → 1. Literature values of the kinetic data required in our model vary depending on the PDE subtype as well as methodical choices made in the experiments. For the diffusive mobility *D* values have been reported in the order of from *D* = 136*μ*m^2^/s [31, 32] while typical values for the absoprtion rate of the PDE4 family are in the order of *k*_*cat*_ ≈ 5s^−1^ and the Michaelis-Menten constant *K*_*m*_ ≈ 2.41*μM* [33, 34]. The radius of the PDE molecule can be estimated from measurements of its crystal structure [35, 36] to be in the order of *R*_*PDE*_ = 2.5nm

Based on these data the absorptive action of the PDE is estimated as *η*_1_ = 7.7 ⋅ 10^−4^. Inserting this value into Eq (14) gives the depth *γ* ≈ 1, which implies that the absorptive action of a single PDE molecule is much (more than 100-fold) too small to lead to any significant nanocompartment. This is in strong disagreement with experiments (s. above) [27], in which the binding curves of the Epac1-camps-PDE4A1 construct provide the depth of the nanocompartments directly.

Even though we used values that are typical for the PDE4A1 subtype used in our experiments, this result is also true if we assume higher values for *k*_*cat*_ that have been reported for other PDE families. For example [37] reported values of up to *k*_*cat*_ = 20s^−1^ for the PDE2 family, which yields *η*_1_ = 30.8 ⋅ 10^−4^.

### Comparison of experimental data with analytical results

We will now compare our model with experimental data from the fusion protein Epac1-camps-PDE4A1 measured in a crude cytosolic preparation (see Fig 2) to get an estimate of the absorptive action *η* of the PDE. The dependence of the sensor signal intensity as a function of the cAMP concentration at the sensor position is revealed by Eq (1). The spatial dependence of the cAMP concentration as a function of the absorptive action is revealed by Eq (12). Combining these equations yields the FRET signal as a function of the absorptive action, external cAMP concentration *c*_0_ = *ρ*_0_/*Km* and inter-spatial PDE-sensor distance *R*_PDE_ + *d* as

$$\begin{array}{c} S=\frac{c_{0}-\frac{1}{2}(\left(1+\eta +c_{0}\right)-\sqrt{{\left(1+\eta +c_{0}\right)}^{2}-4c_{0}\eta })\frac{R_{\text{PDE}}}{R_{\text{PDE}}+d}}{\frac{K_{D}}{K_{m}}+c_{0}-\frac{1}{2}(\left(1+\eta +c_{0}\right)-\sqrt{{\left(1+\eta +c_{0}\right)}^{2}-4c_{0}\eta })\frac{R_{\text{PDE}}}{R_{\text{PDE}}+d}}\;. \end{array}$$ (18)

**Fig 5:**
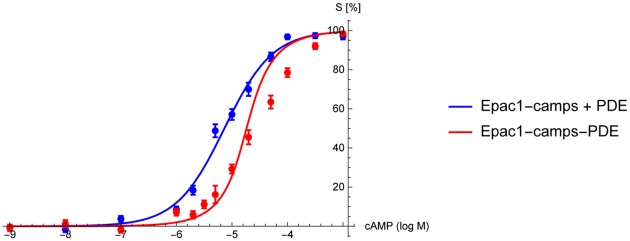
Binding curves of the free sensor protein Epac1-camps + PDE (blue) and the fusion protein Epac1-camps-PDE4A1 (red). The overall PDE activity is equal in both experiments, indicating that the right shift of the binding curve is solely caused by local PDE degradation. The curves were determined by fitting Eq (18) to the corresponding experimental data. The distance between sensor and absorbing enzyme was set to *d* = 0nm as for an ideal sensor-enzyme construct. The fit of the free sensor data (blue) yields the dissociation constant of the sensor *K*_*D*_ = 7.3 ± 0.66*μ*M (goodness of fit: *χ*^2^ = 3.2, *p* = 0.66). From the sensor-enzyme construct (red) we obtain the absorptive action of a PDE *η*_2_ = 6.1 ± 1.4, (*χ*^2^ = 9.6, *p* = 0.08). The flooding of the nanocompartment as predicted by our model could not be observed in the experiments.

We fitted this equation to the experimental data described in the methods section. All fits were performed using Mathematica (Wolfram Research, Inc., Champaign, Illinois, USA). Goodness of fit was assessed using Pearson’s chi squared test. In the experiments (Fig 5) the FRET signal *S* was determined for cAMP concentrations ranging from *ρ*_0_ = 10^−9^M to *ρ*_0_ = 10^−3^M. In control experiments the free sensor protein Epac1-camps (with only basal PDE activity) was measured, i.e. *η* = 0. Note that in this case the concentration-effect curve Eq (18) formally reduces to a simple hyperbola $S=\frac{c_{0}}{K_{D}+c_{0}}$, since *η* = 0, *d* → 0 and then *R*_PDE_ → 0. Fitting this simplified concentration-response curve to Eq (18) revealed the dissociation constant *K*_*D*_ of the sensor as *K*_*D*_ = 7.3 ± 0.66*μ*M, with *χ*^2^ = 3.2, *p* = 0.66 indicating good fit results. In the other experiments the sensor was directly fused to the PDE molecule, i.e. the intermolecular distance *d* was close to zero, where the overall PDE concentration was the same as in the experiments with the free sensor. The shift of the FRET signal curve to the right when the sensor is fused directly to the PDE (see Fig 5) implies a significantly reduced cAMP concentration at the sensor, and, hence, on the reactive PDE surface, when compared to the situation where PDE and Epac1-camps were expressed separatly. We fitted our model (Eq (18)) to the concentration-effect curve measured in the experiments. To avoid overfitting we assumed *d* = 0 and therefore $\frac{R_{PDE}}{R_{PDE}+d}=1$. Further analysis of the mathematical properties of Eq (18) revealed that the fit did not converge for *K*_*m*_, due to low sensitivity at high *K*_*m*_ values. We therefore set *K*_*m*_ to the literature value of *K*_*m*_ ≈ 2.41*μ*M [33, 34]. The fit then yields *η*_2_ = 6.1 ± 1.4, with *χ*^2^ = 9.6, *p* = 0.08. The fit quality is good for lower concentrations of cAMP, but the fitted curve deviates significantly as the cAMP concentration is increased. For high concentrations of cAMP our model predicts that the nanocompartments should be flooded by cAMP and disappear, leading to a steeper slope in the FRET signal. This was not observed in the experiments. It is yet unclear, how the nanocompartments can be maintained even for high concentrations of cAMP.

On the other hand we derived an absorptive action of *η*_1_ = 7.7 ⋅ 10^−4^ from theory in the previous section. The two results differ by up to 4 orders of magnitude. This discrepancy might be explained by the very different experimental setups of the two approaches—in the sensor experiments local nano structures around the PDE might play a significant role in increasing the absorptive action, e.g. by restricting cAMP diffusion. A higher absorptive action in vivo implies either a higher ratio of the catalytic activity to the Michaelis-Menten constant, or a lower diffusion coefficient of cAMP, when compared to literature data. However, as it does not seem plausible that in vivo conditions enhance the enzymatic activity by several orders of magnitude, the reduced diffusive mobility seems to be the most likely option to explain this observation. Other authors come to similar conclusions and suggest reduced diffusive mobility as a key factor in cAMP compartmentation [2, 38].

### Clusters of PDE

**Fig 6:**
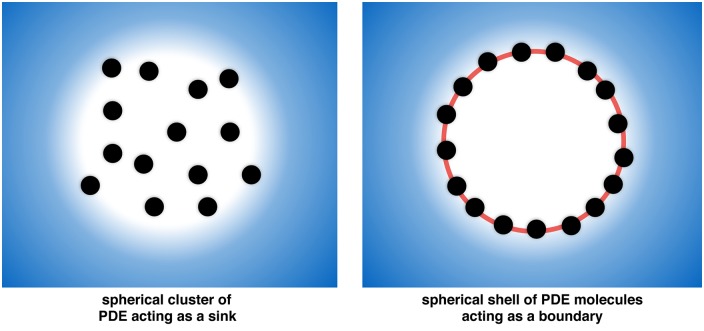
Models of clusters of a degrading enzyme. Left: the degrading enzyme (here PDE) has a homogeneous distribution within a spherical region of diameter *R*_•_. Right: the degrading enzyme forms a thin layer acting as a protective border for the inner region.

A possible explanation for the existence of nanocompartments despite the small absorptive action of a single PDE molecule could be the formation of larger clusters of PDE—as suggested e.g. by Conti et al. [39]—as a possible mechanism of “protecting” larger regions from cAMP. In this section we examine two idealized geometries of such clusters: first a sphere filled with a constant concentration of PDE and second a spherical shell acting as an absorbing border. For these simple geometries we were able to provide analytical solutions of the corresponding diffusion-reaction equations and can thereby provide conditions for the formation of protected regions within such a cluster of PDE—which we will call microcompartments of cAMP. Schematic representations of the two cluster geometries, where PDEs are aggregated within a sphere or on a spherical surface, respectively, are depicted in Fig 6.

### Spherical PDE cluster

The PDE molecules are assumed to be aggregated within a spherical cluster with radius *R*_•_ and there are none outside of this cluster—i.e. absorption only exists for *r* ≤ *R*_•_. We further simplify the diffusion-reaction process by assuming a linear dependence of the absorption rate of a single PDE molecule on the cAMP concentration, which is justified in the low concentration range (*ρ*_*cAMP*_ <<*K*_*m*_). This simplifies the absorption rate of a single PDE molecule (see Eq (4)) to

$$\begin{array}{c} 4\pi R_{\text{PDE}}^{2}j{|}_{r=R_{\text{PDE}}}\approx \frac{k_{cat}}{K_{m}}\rho_{cAMP}{|}_{r=R_{\text{PDE}}} \end{array}$$ (19)

We now focus on the cAMP degradation within the sphere. Here, the average PDE-free volume around a PDE molecule is just the inverse of the density of PDE molecules, *ρ*_*PDE*_, within the sphere, i.e.

$$\begin{array}{c} V_{0}=1/\rho_{\text{PDE}}\;. \end{array}$$ (20)

We assume that the PDEs are packed sufficiently dense so that the cAMP concentration *ρ*_*cAMP*_ is constant within *V*_0_, i.e.

$$\begin{array}{c} \rho_{cAMP}\left(r\right)\equiv \rho_{cAMP}\left(R_{\text{PDE}}\right)\;. \end{array}$$ (21)

This is justified as long as the diffusion time between neighboring PDEs is small when compared to the degradation rate of the PDE. These different time scales allow to replace formally the cAMP degradation at the PDE surface by a constant degradation rate *ξ* at each point within *V*_0_. Self consistently the degradation within *V*_0_, i.e. *ξV*_0_*ρ*_*cAMP*_ must be equivalent to that in Eq (19), i.e. *ξ* is determined as

$$\begin{array}{ccc} \xi & = & \frac{1}{V_{0}}\;\frac{k_{cat}}{K_{m}}\\ & = & \frac{4\pi D\eta R_{\text{PDE}}}{V_{0}} \end{array}$$ (22)

where the latter relation follows from Eq (8). With this degradation rate per volume we may write the temporal evolution of the cAMP concentration in form of a diffusion reaction equation

$$\begin{array}{c} \frac{\partial }{\partial t}\rho \left(x,t\right)=D\Delta \rho \left(x,t\right)-\{\begin{array}{cc} \xi \rho (r,t) & r\leq R_{•}\\ 0 & r>R_{•} \end{array}\;, \end{array}$$ (23)

which accounts for the fact that there is no cAMP degradation outside of the cluster (*ξ* = 0). In the steady state one obtains for the cAMP concentration in-, and around the spherical cluster

$$\begin{array}{c} \rho \left(r\right)=\rho_{0}\{\begin{array}{cc} \frac{\sinh(\zeta \;s)}{\zeta s}\;\cosh{\left(\zeta \right)}^{-1} & s\leq 1\\ 1-\frac{\zeta -\tanh(\zeta )}{\zeta \;s} & s>1 \end{array} \end{array}$$ (24)

with the dimensionless parameter $\zeta =\sqrt{\frac{\xi R_{•}^{2}}{D}}$ and the radius scaled to the cluster radius *r* → *s* = *r*/*R*_•_. So for this cluster geometry *ζ* is analogous to the absorptive action *η* in our model of the single PDE molecule in the way that it is a dimensionless parameter that characterizes the shape of the microcompartment.

With Eq (22) one gets

$$\begin{array}{c} \zeta =\sqrt{\frac{\xi R_{•}^{2}}{D}}=\sqrt{\frac{4\pi \eta R_{•}^{2}R_{\text{PDE}}}{V_{0}}} \end{array}$$ (25)

Or with respect to the number of PDE within the cluster $N_{\text{PDE}}=\frac{4}{3}\pi R_{•}^{3}/V_{0}$,

$$\begin{array}{c} \zeta =\sqrt{3N_{\text{PDE}}\;\eta \frac{R_{\text{PDE}}}{R_{•}}}\;. \end{array}$$ (26)

This gives a simple expression for the shape of a compartment generated by a spherical cluster of a given number of PDE molecules *N*_PDE_ and radius *R*_•_. As shown above we obtain very different values of the absorptive action *η* in the microscopic model—depending on the approach used to determine it. Therefore we also obtain very different values for the absorptive action *ζ* in the mesoscopic model of the spherical cluster.

For *η*_1_ = 7.7 ⋅ 10^−4^ (as derived from the kinetic data found in literature) we obtain

$$\begin{array}{c} \zeta_{1}=\sqrt{N_{\text{PDE}}\frac{6.9\cdot 10^{-3}\text{nm}}{R_{•}}} \end{array}$$ (27)

while *η*_2_ = 6.1 ± 1.4 (as derived from our experimental data) yields

$$\begin{array}{c} \zeta_{2}=\sqrt{N_{\text{PDE}}\frac{109\text{nm}}{R_{•}}} \end{array}$$ (28)

### Conditions for microcompartments within a spherical cluster

In this section we derive conditions under which a spherical cluster of PDE can lead to a significant decrease in the local cAMP concentration. For the depth of the compartment *γ* as defined by Eq (14) we obtain $\gamma =\frac{1}{\cosh(\zeta )}$ or equivalently for a microcomparment of depth *γ*

$$\begin{array}{c} \zeta =\cosh^{-1}(\frac{1}{\gamma }) \end{array}$$ (29)

We can use this equation to estimate number of PDE molecules required to shape a microcompartment of given size *R*_•_ and depth *γ*. If we set *R*_•_ = 100nm and $\gamma =\frac{1}{10}$ then the corresponding number of PDE molecules would be $N_{\text{PDE}}=\frac{\zeta^{2}}{3\;\eta \;R_{\text{PDE}}}R_{•}=130\;000$, for *η*_1_ = 9.2 ⋅ 10^−4^ (from theory) or *N*_PDE_ = 8.5 for *η*_2_ = 6.1 ± 1.4 (from our experiments).

The total number of PDE in e.g. a pulmonary microvascular endothelial cell is estimated to be about *N*_PDE,total_ = 5000 [2]. This would by far not be enough to explain the formation of microcompartments if we assume *η*_1_ = 9.2 ⋅ 10^−4^ as derived from theory.

**Fig 7:**
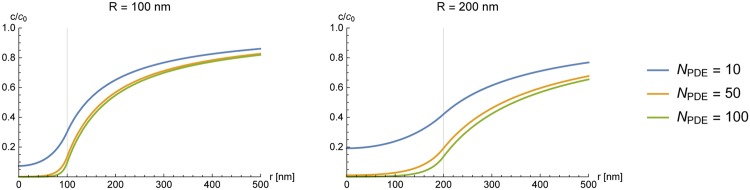
Concentration of cAMP inside a spherical cluster of PDE for different numbers of degrading molecules *N*_PDE_. For both plots the absorptive action of a single PDE was set to *η*_2_ = 6.1 ± 1.4. Left: cluster with radius *R*_•_ = 100nm; to form a microcompartment of this size there required number of PDEs is about *N*_PDE_ = 10. Right: cluster with radius *R*_•_ = 200nm. The depth of the microcompartment is smaller when the number of degrading molecules is kept constant.

In Fig 7 we show the concentration of cAMP in microcompartments of different size and concentration of PDE under the conditions of *η*_2_ = 6.1 ± 1.4 as derived from our experiments.

### Spherical shell PDE cluster

In this section we focus on a second cluster geometry, where the PDE molecules form an absorbing spherical shell in order to protect the enclosed region from cAMP. The question of interest is whether such a cluster of PDE could lead to a significantly decreased cAMP concentration in the inner region of the spherical shell under physiological conditions. Further it helps to understand the impact of cluster geometry on depth and shape of the microcompartment. The mathematical treatment of this case is essentially analogous to the absorbing sphere—therefore we will only discuss the main results in this section. To facilitate analytical treatment of the corresponding diffusion-reaction equation we will again assume a linear relationship between absorption rate and cAMP concentration—as argued in the previous section this approximation is justified in the low concentration range. The corresponding diffusion reaction equation reads

$$\begin{array}{c} \frac{\partial }{\partial t}\rho =D\rho -\chi \;\rho \left(r,t\right)\delta \left(r-R_{{}^{\circ}}\right) \end{array}$$ (30)

with reaction rate constant *χ* > 0 and the delta distribution *δ*(*r* − *R*_°_) restricting absorption is to the shell surface. The steady state solution of this equation is given by

$$\begin{array}{c} \rho \left(r\right)=\{\begin{array}{cc} \rho_{i} & r\leq R_{{}^{\circ}}\\ \rho_{0}-\left(\rho_{0}-\rho_{i}\right)\frac{R_{{}^{\circ}}}{r} & r>R_{{}^{\circ}} \end{array} \end{array}$$ (31)

where the concentration on the inside of the absorbing spherical shell is given by

$$\begin{array}{c} \rho_{i}=\rho_{0}\frac{1}{1+\frac{\chi R_{{}^{\circ}}}{D}} \end{array}$$ (32)

Note that in fact the concentration of cAMP is constant within the boundary of the absorbing spherical shell. From this we also obtain the depth of the microcompartment

$$\begin{array}{c} \gamma =\frac{\rho_{i}}{\rho_{0}}=\frac{1}{1+\frac{\chi R_{{}^{\circ}}}{D}} \end{array}$$ (33)

Analogous to the treatment of the solid sphere we can relate the reaction rate constant *χ* to the number of the absorbing centers *N*_PDE_ on the surface of the spherical shell

$$\begin{array}{c} \chi =\frac{R_{\text{PDE}}\;\eta \;D\;N_{\text{PDE}}}{R_{{}^{\circ}}^{2}} \end{array}$$ (34)

with *η* the absoprtive action of a single molecule of PDE in the microscopic model. Of particular interest is the relationship between the number of absorbing PDE molecules on the shell surface and the “depth” of the compartment *γ*. In the case of a spherical shell it depends on the ratio *N*_PDE_/*R*_°_ as can be derived from Eqs (33) and (34)

$$\begin{array}{c} \gamma =\frac{1}{1+\eta N_{\text{PDE}}\frac{R_{\text{PDE}}}{R_{{}^{\circ}}}} \end{array}$$ (35)

**Fig 8:**
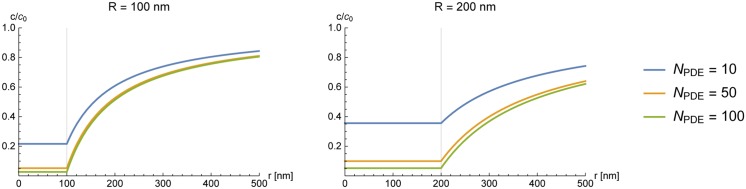
Concentration of cAMP inside a spherical shell cluster of PDE for different numbers of degrading molecules *N*_PDE_. For both plots the absorptive action of a single PDE was set to *η*_2_ = 6.1 ± 1.4. Left: cluster with radius *R*_•_ = 100nm; to form a microcompartment of this size there required number of PDEs is about *N*_PDE_ = 10. Right: cluster with radius *R*_•_ = 200nm. The depth of the microcompartment is smaller when the number of degrading molecules is kept constant.

This leads to very similar findings as in the previous section: to form a microcompartment of depth *γ* and of radius *R*_°_ the required number of PDE is $N_{\text{PDE}}=(\frac{1}{\gamma }-1)\frac{R_{{}^{\circ}}}{\eta \;R_{\text{PDE}}}$. This leads to physiological values only if we assume *η*_2_ = 6.1 ± 1.4 (as obtained from our experiments)—for example *γ* = 0.1, *R*_°_ = 100nm yields: *N*_PDE_ ≈ 25, while assuming *η*_1_ = 7.7 ⋅ 10^−4^ (as obtained from theory) yields *N*_PDE_ ≈ 39 ⋅ 10^4^ for the same compartment (Fig 8).

These numbers for the spherical shell are in the same order of magnitude as the numbers found for the solid sphere in the previous section, which suggests that the cluster geometry has only minor impact on the shape of the compartment. We could further show that the absorptive action *η* of a single PDE molecule has to be in the order of magnitude of *η* ≈ 1 in order to form compartments under physiological conditions. This again suggests either dramatically impeded diffusion speeds or increased absorption rates.

### Summary of mathematical modeling

We sum up the results derived from our mathematical model as follows:

- The ability of a single molecule of PDE to create a nanocompartment can be described by its absoprtive action $\eta =\frac{k_{cat}}{4\pi R_{PDE}DK_{m}}$. Similar parameters can be introduced for clusters of PDE.
- The nanocompartments can be flooded when the concentration of cAMP is increased. The concentration where the compartment is half way flooded is given by $\rho_{1/2}=K_{m}\frac{{\left(1+\eta \right)}^{2}}{1+\eta /2}$
- Given current literature values for diffusive mobility and enzyme parameters, neither a single molecule nor a large cluster of PDE would be sufficient to create cAMP compartments.
- Fitting of the concentration-signal curve of the Epac1-camps-PDE4A1 construct yields *η* = 6.1 ± 1.4—this value is about 10^4^ times higher than the values estimated from theory.

## Discussion

cAMP is a ubiquitous second messenger mediating a myriad of cellular functions. Although it was initially believed that cAMP is uniformly distributed within the cell, a great body of evidence supports the notion that cAMP is compartmentalized. cAMP is produced upon stimulation of a variety of *G*_s_-coupled receptors expressed in a cell and the concept of compartmentation would allow a high degree of spatial and temporal cAMP signaling specificity. Despite the fundamental importance of signal compartmentation, the molecular mechanisms of the generation and dynamics of these compartments are largely unknown.

In this paper we have studied the influence of PDEs in the formation of cAMP nanocompartments combining FRET-based measurements of cAMP concentrations and a mathematical analysis based on PDE activity and cAMP diffusion. To directly assess local cAMP concentrations next to a PDE we have used fusion proteins comprised of the cAMP FRET-sensor Epac1-camps and PDE4A1.

Based on these data, we provide a model for the binding curve of the sensor in such a fusion protein in order to estimate the absorptive action of a single PDE molecule from these measurements (Fig 2). Using this approach we found that the reaction rate required in order to form such a nanocompartment is about three to four orders of magnitude higher than the actual reaction rates of PDE, as long as free diffusible cAMP molecules are assumed. However, the in vivo existence of such nanocompartments is shown by our experimental results (Fig 1) as suggested already in earlier experiments [27]. Moreover, even upon disruption of the cellular environment, the tethered PDE shields the cAMP sensor from cAMP (Fig 2).

A possible explanation of these measurements could be the formation of clusters of PDE that would lead to a decreased concentration of cAMP on a larger scale (which we refer to as microcompartments). Such clusters would need to be formed by stabilizing proteins. In line with this, compartmentation of PDEs by A-kinase anchoring proteins (AKAPs) has been reported by other authors [39–41]. However, even if one considers PDE-anchofing by AKAPs, we found that unphysiological amounts of PDEs would be required to achieve a substantial change in the concentration of cAMP by a cluster of PDE’s. Therefore further yet unknown mechanisms have to contribute to the compartmentation of cAMP in living cells.

Several other groups have reported mathematical models of cAMP degradation on a large scale [6, 7] as well as numerical simulations [2, 38] and in line with these studies, our data strongly support the finding that PDE activity alone should be insufficient to explain compartmentation of cAMP on a nanometer scale.

Therefore we suggest that cAMP diffusion within the nanodomain must be restricted. Recent studies have provided first evidence that cAMP diffusion within cells may be 1 order of magnitude (ca. 10*μ*m^2^/s) slower than previously anticipated [26, 42]. However, based on our calculation, this diffusion speed is still not sufficient to create a nanodomain in a cytosolic environment. Future studies need to reassess the heterogeneity of cAMP diffusion within cells. Moreover, the mechanisms which restrict cAMP diffusion are entirely unknown. One possible physical parameter which could potentially restrict cAMP diffusion is locally increased microviscosity [38, 42, 43]. This could lead to areas of impeded diffusion and therefore dramatically increased values for the absorptive action *η* in our models. However it is yet unclear, whether areas of highly impeded diffusion coincide with the localization of PDE enzymes.

Taken together, we have shown the existence of nanodomains of low cAMP in cells. Our data suggest that PDEs are only capable of establishing such domains when the diffusion of cAMP is restricted. Further experiments will investigate the role of inhomogeneous diffusion in the formation of cAMP nanocompartments and will aim to measure the spatial extent and shape of the cAMP nanocompartments.

## Supporting information

S1 Fig Fluorescence spectra of Epac1-camps and Epac1-camps-PDE4A1. Shown are fluorescence emission spectra of cytosolic fractions of HEK-TsA cells expressing Epac1-camps (a) and Epac1-camps-PDE4A1 (b) obtained in a 10mM TRIS − HCl/10mM MgCl_2_ buffer. A cAMP-dependent decrease in the YFP/CFP ratio is demonstrated.
(TIF)

S2 Fig Tethering of PDE4A1 to Epac1-camps does not alter its apparent cAMP affinity. Concentration-effect curve of cAMP-induced changes of the FRET ratio of the cAMP sensor Epac1-camps-PDE4A1 (D352A) in cytosolic preparations of transiently transfected HEK-TsA cells (black curve). The concentration-effect curves of Epac1-camps (grey) and Epac1-camps-PDE4A1 (red) are shown for comparison. The apparent affinity (pEC_50_) of Epac1-camps-PDE4A1 (D352A) is 5.56 ± 0.08(= 2.7*μ*M) and thereby not different from Epac1-camps (see manuscript text). Data are means ± s.e.m. of three independent experiments carried out with 2-3 repetitions.
(TIF)

S3 Fig Adjustment of PDE4A1 protein levels based on catalytic activities calculated from transient FRET changes. (a-c) Representative real-time, in vitro FRET measurements of cytosolic preparations of HEK-TsA cells transiently expressing the indicated constructs. Addition of 100*μ*M cAMP (red arrow) leads to a decrease in FRET (YFP/CFP) ratio due to binding of cAMP to the sensors. (a) In case of Epac1-camps-PDE4A1 the FRET change is transient and increases to the basal FRET ratio after ≈350s due to PDE activity. (b) At the same expression level the FRET change is not transient in cytosolic preparations only expressing Epac1-camps indicating that endogenous PDE activity is negligible. (c) Separate expression of Epac1-camps and PDE4A1: the amount of PDE4A1 cytosol was adjusted to the same catalytic activity (Δ*τ* as surrogate parameter) as measured with Epac1-camps-PDE4A1. (d) Δ*τ* values in cytosolic preparations expressing Epac1-camps-PDE4A1 (red) or Epac1-camps + PDE4A1 (blue) are not significantly different (P = 0.99, according to an unpaired t-test). Data in (d) are means ± s.e.m. of 4 independent experiments, representatives of which are shown in (a) and (c).
(TIF)
