# Supplementary figures and images for: Experimental and mathematical analysis of cAMP nanodomains
Source: PLoS One. 2017 Apr 13;12(4):e0174856. doi: 10.1371/journal.pone.0174856 (PMC5391016; doi:10.1371/journal.pone.0174856)

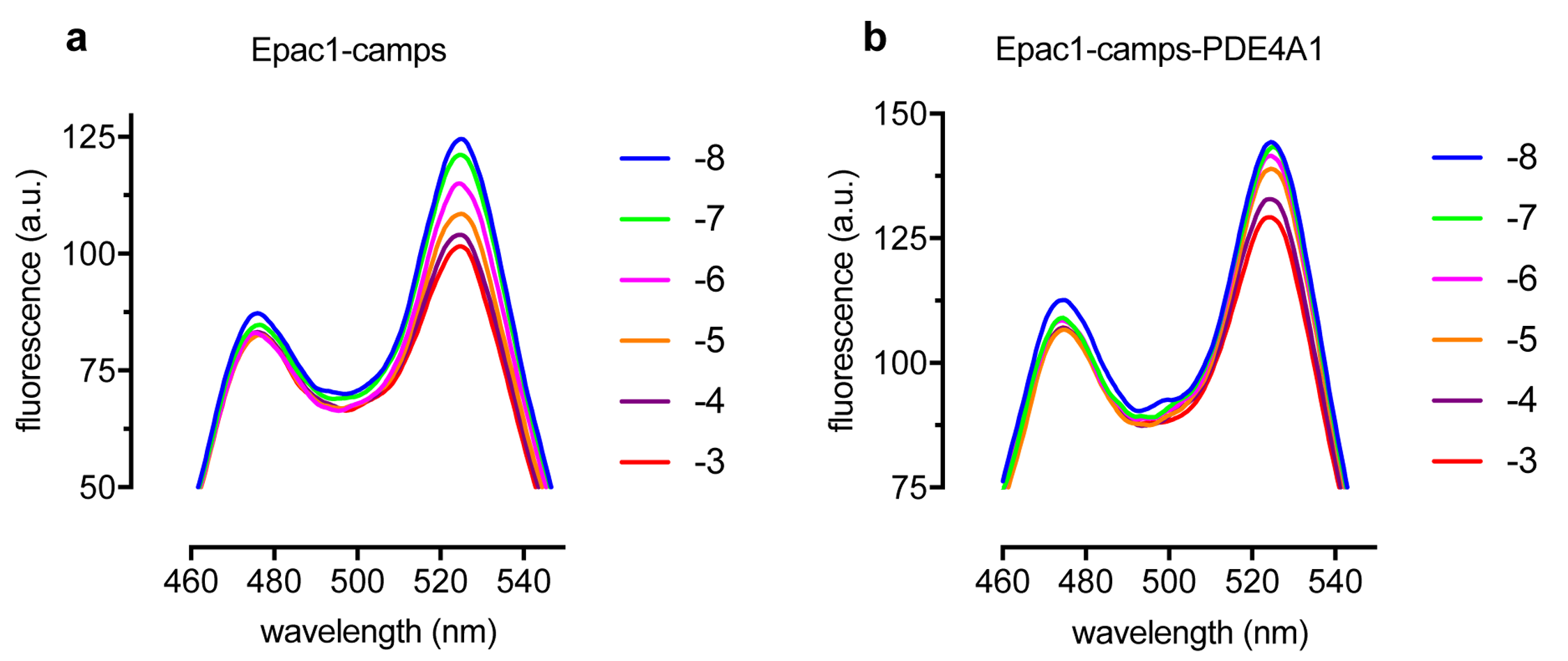

Supplement: S1 Fig — Shown are fluorescence emission spectra of cytosolic fractions of HEK-TsA cells expressing Epac1-camps (a) and Epac1-camps-PDE4A1 (b) obtained in a 10mM TRIS − HCl/10mM MgCl2 buffer. A cAMP-dependent decrease in the YFP/CFP ratio is demonstrated. (TIF) [file pone.0174856.s001.tif]

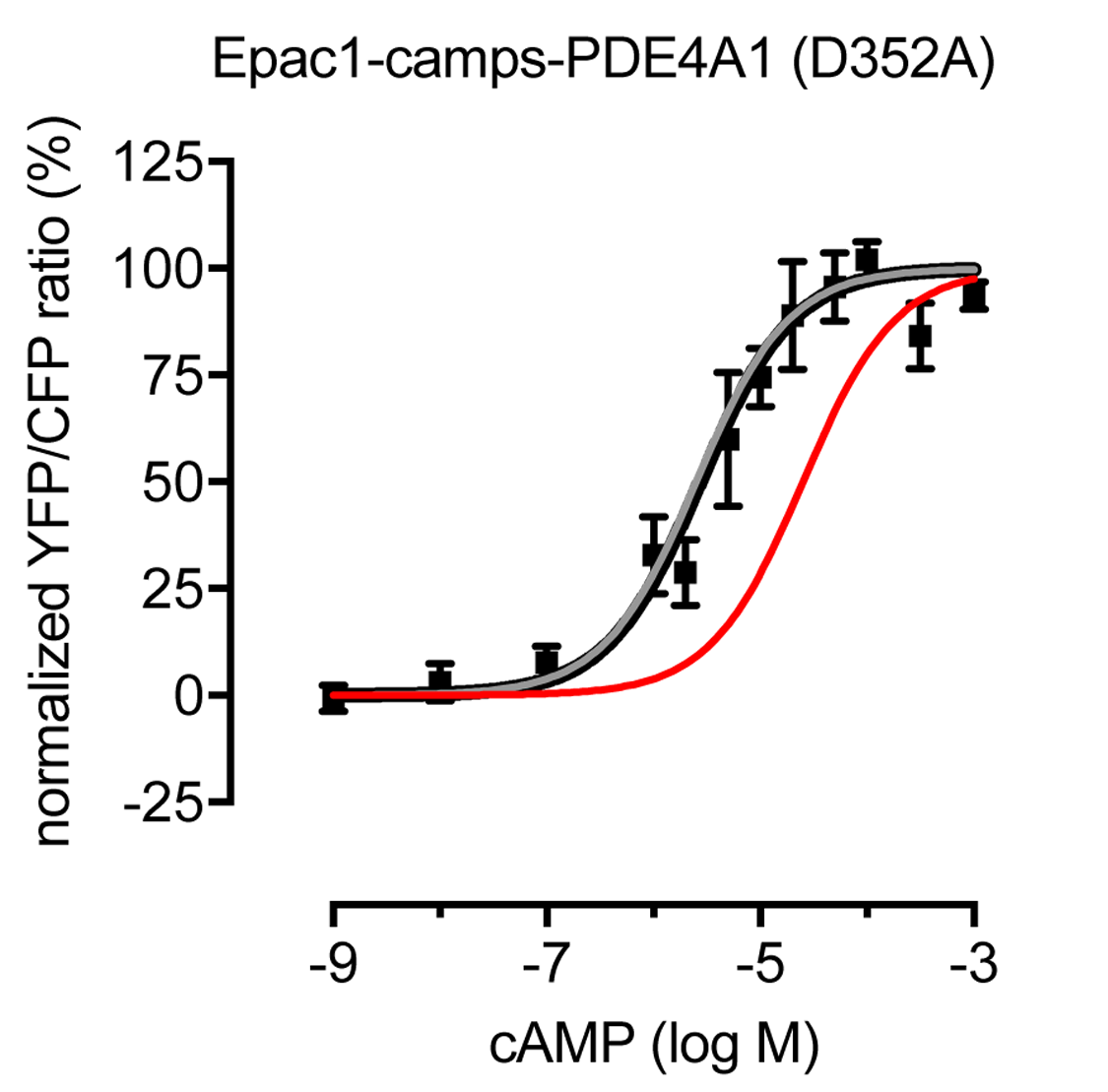

Supplement: S2 Fig — Concentration-effect curve of cAMP-induced changes of the FRET ratio of the cAMP sensor Epac1-camps-PDE4A1 (D352A) in cytosolic preparations of transiently transfected HEK-TsA cells (black curve). The concentration-effect curves of Epac1-camps (grey) and Epac1-camps-PDE4A1 (red) are shown for comparison. The apparent affinity (pEC50) of Epac1-camps-PDE4A1 (D352A) is 5.56 ± 0.08(= 2.7μM) and thereby not different from Epac1-camps (see manuscript text). Data are means ± s.e.m. of three independent experiments carried out with 2-3 repetitions. (TIF) [file pone.0174856.s002.tif]

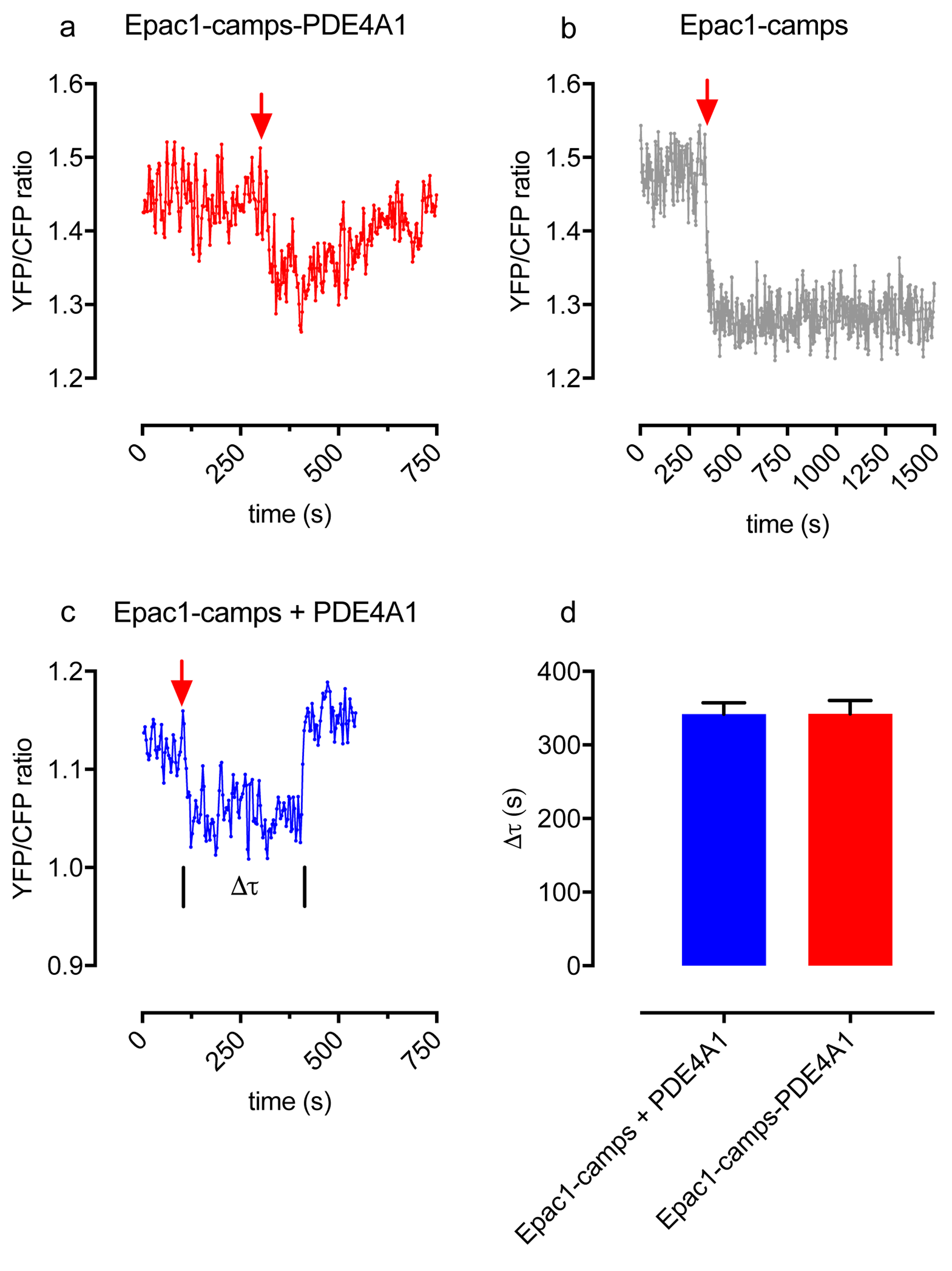

Supplement: S3 Fig — (a-c) Representative real-time, in vitro FRET measurements of cytosolic preparations of HEK-TsA cells transiently expressing the indicated constructs. Addition of 100μM cAMP (red arrow) leads to a decrease in FRET (YFP/CFP) ratio due to binding of cAMP to the sensors. (a) In case of Epac1-camps-PDE4A1 the FRET change is transient and increases to the basal FRET ratio after ≈350s due to PDE activity. (b) At the same expression level the FRET change is not transient in cytosolic preparations only expressing Epac1-camps indicating that endogenous PDE activity is negligible. (c) Separate expression of Epac1-camps and PDE4A1: the amount of PDE4A1 cytosol was adjusted to the same catalytic activity (Δτ as surrogate parameter) as measured with Epac1-camps-PDE4A1. (d) Δτ values in cytosolic preparations expressing Epac1-camps-PDE4A1 (red) or Epac1-camps + PDE4A1 (blue) are not significantly different (P = 0.99, according to an unpaired t-test). Data in (d) are means ± s.e.m. of 4 independent experiments, representatives of which are shown in (a) and (c). (TIF) [file pone.0174856.s003.tif]
